# The Relationship Between Autism and Pitch Perception is Modulated by Cognitive Abilities

**DOI:** 10.1007/s10803-023-06075-7

**Published:** 2023-08-29

**Authors:** Jia Hoong Ong, Chen Zhao, Alex Bacon, Florence Yik Nam Leung, Anamarija Veic, Li Wang, Cunmei Jiang, Fang Liu

**Affiliations:** 1 School of Psychology and Clinical Language Sciences, University of Reading, Reading, UK; 2 Department of Psychology, School of Social Sciences, Nottingham Trent University, Nottingham, UK; 3 Music College, Shanghai Normal University, Shanghai, China

**Keywords:** Pitch, Cognitive, Individual differences, Matching, Autism

## Abstract

Previous studies reported mixed findings on autistic individuals’ pitch perception relative to neurotypical (NT) individuals. We investigated whether this may be partly due to individual differences in cognitive abilities by comparing their performance on various pitch perception tasks on a large sample (*n* = 164) of autistic and NT children and adults. Our findings revealed that: (i) autistic individuals either showed similar or worse performance than NT individuals on the pitch tasks; (ii) cognitive abilities were associated with some pitch task performance; and (iii) cognitive abilities modulated the relationship between autism diagnosis and pitch perception on some tasks. Our findings highlight the importance of taking an individual differences approach to understand the strengths and weaknesses of pitch processing in autism.

Pitch is ubiquitous in our everyday listening experiences (Gaver, 1993). Embedded in countless sound events, pitch carries the frequency information of sound that enables us to understand language, appreciate music, and navigate our environment (Patel, 2008; Xu, 2005; Zatorre & Baum, 2012). Despite its necessity, the ability to process pitch in different stimuli varies across individuals, with moderating factors including intelligence (Acton & Schroeder, 2001; Deary et al., 1989; Helmbold et al., 2006; Raz et al., 1987; Spearman, 1904; Watson, 1991), age (Fancourt et al., 2013; Lamont, 1998), memory (Moore et al., 2007; Tillmann et al., 2016), music aptitude (Bidelman et al., 2013; Lynn et al., 1989; Schellenberg & Weiss, 2013; Wong et al., 2007), tone language background (Bidelman et al., 2013; Pfordresher & Brown, 2009), as well as other individual differences as seen in atypical populations such as congenital amusia, a neurodevelopmental disorder of pitch processing (Ayotte et al., 2002; Liu et al., 2010, 2012; Vuvan et al., 2015). Thus, comparing pitch processing across different groups and samples requires consideration of various background measures, which could confound the findings even in matched case-control studies (Pearce, 2016).

The present study focuses on pitch processing among individuals with and without autism spectrum disorder (ASD) (American Psychiatric Association, 2013), hereafter termed autistic^1^ and neurotypical (NT) individuals, respectively. A deeper understanding of the relationship between autism and pitch perception will help shed light on various autistic experiences. Firstly, pitch plays an important role in communication, such as in expressing affective and linguistic prosody, both of which autistic individuals are said to show differences in (O’Connor, 2012). It may be the case that these differences partly stem from atypical basic pitch perception generally. Secondly, autistic individuals are reported to have higher prevalence of auditory hypersensitivity (Talay-Ongan & Wood, 2000; Tavassoli et al., 2014) and find high-pitched sounds distressing (Landon et al., 2016; Robertson & Simmons, 2015). What is unclear, however, is whether this reflects a physiological or psychological difference. Moreover, if autistic individuals show a dissociation in pitch perception ability across domains, such as preserved or even enhanced musical pitch perception, but atypical linguistic pitch perception as reported in various studies (Jiang et al., 2015; Schelinski & von Kriegstein, 2019), then this will have implications for using music for therapeutic purposes such as music-assisted language programmes or music therapy (Simpson & Keen, 2011; Williams et al., 2021).

Previous auditory research reported superior pitch perception ability among autistic individuals in the discrimination of simple pure tones (Bonnel et al., 2003, 2010; Eigsti & Fein, 2013) and more complex stimuli such as speech, nonwords, and tone analogues (Heaton, Hudry et al., 2008) compared to NT individuals. Their supposedly superior ability extends to the music domain too, in which autistic individuals outperformed NT individuals in identifying pitch direction of piano tones (Heaton, 2005) as well as discriminating and remembering melodies (Stanutz et al., 2014). Subsequent research in this area, however, revealed that the advantage in pitch perception among autistic individuals is not as straightforward as it seems. The advantage may be more evident under certain conditions, such as when the stimuli are simple tones (Ouimet et al., 2012) or nonspeech tones (Yu et al., 2015), and may be task-dependent (e.g., the advantage in melodic contour perception was observed using an identification task but not a discrimination task) (Jiang et al., 2015). Moreover, the advantage may only exist within certain subgroups among autistic individuals, such as younger children (Mayer et al., 2016); those with an autism, but not Asperger’s, diagnosis (Bonnel et al., 2010); and those with delayed language onset (Heaton, Hudry et al., 2008; Jones et al., 2009) (though contradictory findings that autistic individuals *without* delayed language onset showed enhanced pitch perception have also been shown (Cheng et al., 2017)).

Further complicating the picture is the existence of contradictory findings. Some studies reported that autistic individuals do not differ in their pitch perception ability relative to NT individuals either in ‘low level’ pitch perception tasks such as those that involve discriminating or identifying simple pure tones (Altgassen et al., 2005; Chowdhury et al., 2017; Germain et al., 2019; Globerson et al., 2015) or in tasks that measure musical pitch perception such as the Montreal Battery of Evaluation of Amusia (MBEA) that involves discriminating pairs of melodies and recognition memory of these melodies (Jamey et al., 2019; Schelinski & Roswandowitz, 2017). In fact, some studies reported that autistic individuals have *impaired* pitch perception relative to NT individuals in various pitch tasks such as in tasks involving ‘low level’ pitch perception (Bhatara et al., 2013; Kargas et al., 2015), speech prosody (Jiang et al., 2015), and in musical tasks such as the MBEA (Sota et al., 2018).

It is thus clear from the literature that the findings are mixed. Reviews conducted in this field of research have generally concluded that methodological differences such as stimulus type, stimulus domain, and task complexity contribute to these mixed findings (Haesen et al., 2011; O’Connor, 2012). Furthermore, previous studies generally have a small sample size in their group comparison (with each group consisting of *n* = 20 or less, typically) and so may lack the statistical power necessary to detect group differences in pitch perception. We argue that, in addition to those factors, another contributing factor to the mixed findings is individual differences in cognitive abilities that have rarely been examined in auditory research but have been shown to be involved in pitch perception nonetheless (Chowdhury et al., 2017; Hou et al., 2014; Spearman, 1904). Though rarely examined directly, there are several reasons to believe individual differences in cognitive abilities may partly explain the mixed findings between autistic and NT individuals in pitch perception. Firstly, from previous research, group differences in pitch perception were usually observed when the groups were not matched on verbal IQ (VIQ) (Eigsti & Fein, 2013) or nonverbal IQ (NVIQ) (Yu et al., 2015) whereas no differences in pitch perception were observed when the groups were matched on either of those cognitive abilities (Chowdhury et al., 2017; Germain et al., 2019; Globerson et al., 2015; Jones et al., 2009; Schelinski & Roswandowitz, 2017), suggesting that group differences may be due to differences in cognitive abilities rather than autism diagnosis per se. Secondly, autistic individuals may rely on certain cognitive abilities that NT individuals do not when completing the same pitch perception task (Jamey et al., 2019; Kargas et al., 2015; Mayer et al., 2016), implying that cognitive abilities may modulate pitch perception differently between groups. However, the modulating role of cognitive abilities in pitch perception between autistic and NT individuals remains speculative as most studies do not formally assess the role of cognitive abilities in pitch perception (i.e., cognitive abilities are typically not included as predictors of pitch perception), and those that do usually infer the differential modulating role of cognitive abilities using separate by-group models and/or correlations (i.e., without a Group × Cognitive Ability interaction). The present study will thus address this directly by examining whether there are any group differences in pitch perception when cognitive abilities are not vs. when they are considered.

In case-control designs common in autism research, it is important to match the cases (e.g., autistic participants) with the controls (e.g., NT participants) on potentially confounding factors (e.g., age, gender, etc.) to isolate the factor of interest (e.g., autism diagnosis). This can either be done at the group level (which may result in unequal sample sizes between the groups but are nonetheless statistically similar in the confounding factors when compared group-wise) or at the individual level (matching participants individually on the confounding factors to ensure that they are statistically similar, and by definition, would result in an equal sample size between the groups). Both types of matching are seen in autism research (e.g., group-level matching: (Chowdhury et al., 2017; Eigsti & Fein, 2013; Jones et al., 2009); individual-level matching: (Heaton, 2005; Schelinski & Roswandowitz, 2017)), though group-level matching is arguably more common given its feasibility. It is not clear, however, whether the different types of matching procedure may affect the likelihood of observing group differences. We reason that group-level matching may increase the likelihood of higher variability in the predictors among the participants, as there is more tolerance of variability on the measures being matched. This may introduce more noise, and thus mask any subtle group difference. Individual-level matching, conversely, may result in a ‘purer’ comparison of the factor of interest at the cost of lower variability in the other predictors, as the cases and the controls may become ‘too similar’ in every other aspect. In the present study, as an exploratory aim, we also examined whether the types of matching procedure will affect the pattern of results. Specifically, we analysed our data using two sets of samples: in one set, we matched all our autistic and NT participants at the group level, whereas in the other, a subset of those participants was matched individually in age (± 5 years) and gender^2^.

In summary, we examined: (i) whether there are any group differences in pitch perception among autistic and NT individuals; (ii) whether pitch perception is related to cognitive abilities; and (iii) if so, whether cognitive abilities modulate the relationship between pitch perception and autism. We approached these research questions with autistic and NT participants from a diverse age range (children to adults) and using various pitch and cognitive tasks. Unlike previous studies that typically employ just one pitch task, we used several pitch tasks that have different task requirements (identification or discrimination) and use a diverse range of stimuli, ranging from simple pure tones to more complex piano and speech tones to musical melodies, to measure participants’ pitch ability holistically. The cognitive abilities examined in this study were receptive verbal ability (as a proxy of verbal IQ) and nonverbal IQ (NVIQ), which are important indicators of intellectual disability; as well as verbal and nonverbal short-term memory, which may be important for pitch tasks involving speech and nonspeech materials, respectively. As an exploratory objective, we also assessed whether different patterns of results would be obtained using two different matching procedures (group-level vs. individual-level matching).

## Method

### Participants

A total of 164 British English-speaking individuals, whose age ranged between 7 and 57, participated in the study. Fifty-nine of the participants were in the ASD group as they have received a clinical diagnosis of ASD^3^ by a licensed clinician whereas the others were in the NT group. Confirming their diagnosis, the ASD group had significantly higher autistic traits as measured using the autism-spectrum quotient (AQ) (Auyeung et al., 2008; Baron-Cohen et al., 2001, 2006) than the NT group^4^ (comparison was conducted for three age groups separately following the AQ test protocol; children: ASD (*n* = 18, M = 93.00, SD = 25.54) vs. NT (*n* = 20, M = 46.50, SD = 18.63), *t*(36) = 6.46, *p* < .001; adolescents: ASD (*n* = 12, M = 35.67, SD = 6.23) vs. NT (*n* = 16, M = 17.25, SD = 7.47), *t*(26) = 6.92, *p* < .001; adults: ASD (*n* = 29, M = 36.93, SD = 8.37) vs. NT (*n* = 67, M = 15.43, SD = 7.54), *t*(94) = 12.40, *p* < .001). The two groups, however, did not differ in their age, musical training experience, or cognitive abilities (see Table 1 below).

**Table 1:** Comparison of means (standard deviations in parentheses) of age, years of musical training, and cognitive abilities between the ASD and the NT group

	ASD (40 M + 19 F)	NT (50 M + 55 F)	Group comparison
Age	21.59 (14.61)	20.97 (11.41)	*t*(162) = 0.30, *p* = .763
Years of Musical Training	3.55 (5.19)	3.77 (4.82)	*t*(162) = 0.27, *p* = .790
ROWPVT-4	113.73 (17.57)	114.63 (17.01)	*t*(162) = 0.32, *p* = .748
Raven’s SPM	56.86 (29.87)	52.90 (30.99)	*t*(162) = 0.80, *p* = .428
Digit Span	6.25 (1.45)	6.62 (1.28)	*t*(162) = 0.95, *p* = .343
Corsi Block	5.59 (1.45)	6.05 (1.37)	*t*(162) = 1.26, *p* = .209

*Note*: M = Male; F = Female; ROWPVT-4 = Receptive One-Word Picture Vocabulary Test, 4th Edition; Raven’s SPM = Raven’s Standard Progressive Matrices

In addition to the overall comparison, we also compared the two groups of individually-matched participants on age, years of musical training, and cognitive abilities on each pitch task separately given that not all participants completed the tasks (due to time constraint from the participant and/or due to some tasks introduced later than others) or met the criterion (see Pitch Tasks subsection below). Similar to the findings from group-level matching, we found no group differences in their demographic characteristics and cognitive abilities (see Supplementary Section S2).

### Cognitive Tasks

#### Receptive One-Word Picture Vocabulary Test (ROWPVT-4)

The ROWPVT-4 (Martin & Brownell, 2011), a pen-and-paper test on receptive vocabulary was taken as an indirect measure for verbal IQ for the present study. Participants were presented with a word auditorily on each trial, and they had to select one of four images that is the most appropriate for that word. Following the test protocol, we first established their basal score, and the task was terminated after participants had at least six incorrect trials within the last eight trials (i.e., their ceiling score). Their raw scores were converted to age-appropriate standard scores.

#### Raven’s Standard Progressive Matrices

The Raven’s Standard Progressive Matrices (Raven et al., 1998) is a 60-trial, pen-and-paper test that measures nonverbal IQ. On each trial, participants were shown an incomplete figure, and they had to complete the figure by choosing one of six to eight options. Participants’ raw scores were converted to age-appropriate percentiles.

#### Digit Span

The digit span, implemented on the Psychology Experiment Building Language (PEBL) Test Battery software (Mueller & Piper, 2014), is a measure of verbal memory. On every trial, participants heard a list of numbers, and they were instructed to repeat the list back to the experimenter. The length of the list increased as the task progressed. Participants’ final scores were the length of the longest list recalled, which were then centred by their age group (defined arbitrarily as the following: children: 7–11 years old; adolescents: 12–15 years old; young adults: 16–39 years old; and middle-aged adults: 40–59 years old).

#### Corsi Block

The Corsi Block task is a measure of nonverbal memory and the version used in the present study was taken from the PEBL software (Mueller & Piper, 2014). On every trial, participants saw a series of squares light up in a sequence, and they had to recall the sequence. The sequence length increased as the task progressed. Similar to the Digit Span task, participants’ final scores were the length of the longest sequence recalled, which were then centred by their age group.

### Pitch Tasks

#### Pitch Detection

The Pitch Detection task, as used in previous studies (Liu et al., 2010, 2012), measures participants’ sensitivity to detect a glide in pitch contour. On every trial, participants heard three 500 Hz pure tones of 600 ms—two of which had a flat contour and one had either an upward or a downward glide—and they were instructed to choose the odd-one-out, which was either the first or the third tone. An adaptive tracking procedure with a two-down-one-up staircase method was used: starting with 6 semitones as the excursion size of the first gliding tone, the step size reduced by 1 semitone, and then by 0.1 semitones after four reversals, and then by 0.02 semitones after eight reversals. The task terminated after 14 reversals and participants’ thresholds were defined as the mean excursion glide value of the last six reversals. Participants were given practice trials prior to the start of the task and they only progressed to the actual task if they had all the practice trials correct. To ensure they understood the task instructions and attentiveness, we only included participants who had the first five test trials correct.

#### Pitch Direction

The Pitch Direction task, taken from previous studies (Liu et al., 2010, 2012), measures participants’ sensitivity to the direction of gliding tones. The task was similar to the Pitch Detection task, with the exception that the stimuli consisted of three gliding tones: two that glided in one direction and the ‘odd-one-out’ in another.

#### Speech Discrimination

The Speech Discrimination task measures participants’ discrimination of pairs of speech syllables differing in their pitch (Liu et al., 2017). Specifically, on every trial, participants heard a pair of /ma/ syllables, one of which was the standard (131 Hz) and the other was the target that was higher in pitch (the order of presentation was randomised between each pair), and participants decided which of the two tones was higher in pitch. The target stimuli deviated from the standard between 0.01 semitones and 12 semitones in 63 steps. An adaptive tracking procedure with a two-down-one-up staircase method was used, and the task terminated after 14 reversals. Participants’ discrimination scores were defined as the mean pitch intervals in the last six reversals. Prior to the task, participants completed a series of practice trials, during which they needed to achieve 100% accuracy before progressing. Similar to the Pitch Detection task, we only included participants who had the first five test trials correct to ensure they understood the task instructions and attentiveness.

#### Piano Discrimination

The Piano Discrimination task (Liu et al., 2017) was similar to the Speech Discrimination task, with the exception that instead of speech syllables, the stimuli consisted of piano tones. Importantly, the same range of frequencies was used as in the Speech Discrimination Task.

#### Musical Pitch Perception

Musical pitch perception was assessed using the Montreal Battery of Evaluation of Amusia (MBEA) (Peretz et al., 2003) for participants aged 16 and above, or the shortened version, the Montreal Battery of Evaluation of Musical Abilities (MBEMA) (Peretz et al., 2013) for those aged less than 16 years old. For the present study, we only analysed the pitch composite, which consists of participants’ mean accuracy on three pitch subtests. In each subtest, participants were presented with pairs of melodies, and they were asked to identify whether each pair was the same or different. The first subtest was Scale, wherein a ‘different pair’ melody had a deviated note that was out-of-tune relative to the scale of the melody. The second and third subtests were Contour and Interval, respectively, wherein a ‘different pair’ melody had a deviated note that was in tune, but the note violated the global melodic contour in the former and had a different interval size in the latter.

### Procedure

Participants completed all the tasks as part of a test battery either over one session with multiple breaks or over multiple sessions. The tasks were presented to the participants in a random order. Participants received monetary compensation or course credit for their participation. Prior to the start of the first task, participants or their caregivers (for participants aged below 16 years old) gave their written informed consent. The study protocol was reviewed and approved by the University Research Ethics Committee (UREC) at the University of Reading.

#### Data Analysis

We analysed the results for each pitch task using two sets of samples: one that involves autistic and NT participants that have been matched at the group level (hereafter ‘group-level sample’) and another that involves matching participants individually (hereafter ‘individual-level sample’).

We first conducted principal component analysis (PCA) to reduce the components of cognitive abilities using *prcomp*() function in R (R Core Team, 2021). This was done on the entire sample for the group-level sample, and on the sample for each pitch task for the individual-level sample. The cognitive scores were *z*-transformed prior to the PCA. In all cases, a three-factor solution was deemed acceptable, explaining at least 86.69% of the variance. Based on the factor loadings, the factors were labelled as “Intelligence”, “Verbal Memory”, and “Nonverbal Memory” (see Supplementary Section S3 for the factor loadings). Note that in cases were the factor loadings were negative—for example, the “Intelligence” component for the Group-level sample– this was inverted to facilitate interpretation of the component, i.e., more positive scores on that component indicates higher performance of that construct.

For each pitch task, consistent with previous studies (Heaton, Williams et al., 2008), to explore whether there are any subgroups within each group, we compared the number of autistic and NT participants who were considered as high-performers (defined as scoring at or above the upper quartile) and those who were not high-performers using a chi-squared test.

To examine the association between the different predictors and pitch perception, we conducted a series of multiple linear regressions for each pitch task using the *lm*() function in R. Specifically, for each pitch task, we modelled a ‘reduced model’, which consisted only of Age (a continuous predictor, which was mean-centred), Group (a categorical predictor, which was effect-coded) and Age × Group interaction to examine whether there are any group differences in each pitch task. Age was included as previous studies have reported increased pitch perception ability with age, at least among neurotypicals (Mayer et al., 2016). Then, for the same pitch task, we modelled a ‘full model’, which included the same predictors as the ‘reduced model’ as well as the three components obtained from the PCA (Intelligence, Verbal Memory, and Nonverbal Memory; all continuous predictors, which have been mean-centred as a result of PCA) and Years of Musical Training (a continuous predictor that was centred by age group: children: 7–11 years old; adolescents 12–15 years old; young adults: 16–39 years old; and middle-aged adults: 40–59 years old) and all interactions involving Age and Group with each of the predictors (e.g., Age × Intelligence, Group × Intelligence, Age × Group × Intelligence, Age × Verbal Memory, etc.). Musical training experience was included as previous studies found a positive relationship between musical training and pitch perception (Posedel et al., 2012). Thus, for each pitch task, we examined four models: reduced and full models using data from the group-level sample as well as reduced and full models using data from the individual-level sample. Model fit between the reduced and full models within each sample were also compared using the Akaike Information Criterion (AIC) with the *AIC*() function. Lower AIC values indicate a better model fit. Consistent with previous studies, pitch thresholds for the pitch detection, pitch direction, speech discrimination, and music discrimination tasks were log-transformed (Liu et al., 2016). Effect sizes for each predictor were estimated using partial eta-squared (η_p_^2^) using the *effectsize*() function in the *effectsize* package (Ben-Shachar et al., 2020). Interactions, if any, were examined using *interact_plot*() function in the *interactions* package (Long, 2020).

## Results

The descriptive statistics for the performance on each pitch task by group and sample type are displayed in Table 2. For brevity, we only report the finding summary with respect to the research questions for all the models that we conducted here (for more detailed information about the results, please refer to Supplementary Section S4).

**Table 2:** Descriptive statistics (number of participants, means, and standard deviation) for each of the pitch task by group and sample type

	Group-level sample				Individual-level sample			
	ASD		NT		ASD		NT	
	*n*	M (SD)	*n*	M (SD)	*n*	M (SD)	*n*	M (SD)
Pitch Detection	45	0.34 (0.48)	84	0.29 (0.65)	40	0.35 (0.50)	40	0.19 (0.10)
Pitch Direction	40	0.69 (0.82)	84	0.43 (0.79)	37	0.67 (0.79)	37	0.33 (0.37)
Speech Discrimination	31	3.57 (4.25)	68	2.52 (3.43)	25	3.65 (4.41)	25	1.26 (2.74)
Piano Discrimination	30	3.33 (4.14)	60	3.20 (4.17)	25	2.90 (3.90)	25	2.40 (3.53)
Musical Pitch Perception	59	79.72% (12.55%)	105	83.88% (10.68%)	53	79.85% (12.95%)	53	84.23% (11.51%)

To address whether there are any group differences in the various pitch perception tasks, Table 3 summarises significant main effects and/or interactions involving the Group predictor in the reduced models for both the group-level and individual-level samples, and the direction of the effect.

**Table 3:** Summary of group differences in each pitch perception task as found in the reduced models for group-level and individual-level samples

Pitch Perception task	Group-level sample	Individual-level sample
Pitch Detection	No group difference	No group difference
Pitch Direction	ASD higher threshold than NT	ASD higher threshold than NT
Speech Discrimination	No group difference	ASD higher threshold than NT
Piano Discrimination	No group difference	No group difference
Musical Pitch Perception	ASD less accurate than NT	No group difference

To examine whether musical training and cognitive abilities are involved in the various pitch perception tasks, Table 4 below summarises significant main effects and/or interactions involving the predictors Years of Musical Training, Intelligence, Verbal Memory, and Nonverbal Memory in the full models for both the group-level and individual-level samples.

**Table 4:** Summary of the involvement of musical training and cognitive abilities in each pitch perception task as found in the full models for group-level and individual-level samples

Pitch Perception task	Group-level sample	Individual-level sample
Pitch Detection	Intelligence, Verbal Memory	Intelligence
Pitch Direction	Intelligence, Verbal Memory	Intelligence
Speech Discrimination	Verbal Memory, Musical Training	None
Piano Discrimination	Intelligence, Musical Training	Intelligence, Musical Training, Nonverbal Memory
Musical Pitch Perception	Intelligence, Verbal Memory	Intelligence, Verbal Memory

Finally, to determine whether there are any group differences in various pitch perception task after taking musical training and cognitive abilities into account, Table 5 displays the presence or absence of significant main effects and/or interactions involving Group in the reduced and full models for both the group-level and individual-level samples.

**Table 5:** Summary of the presence (check) or absence (cross) of significant Group main effect and/or interactions involving Group in each pitch perception task as found in the reduced and full models for group-level and individual-level samples

	Group-level		Individual-level	
Pitch Perception task	Reduced	Full	Reduced	Full
Pitch Detection	✗	✓﻿	✗	✓﻿
Pitch Direction	✓	✓﻿	✓﻿	✗
Speech Discrimination	✗	✗	✓﻿	✗
Piano Discrimination	✗	✗	✗	✓﻿
Musical Pitch Perception	✓﻿	✓﻿	✗	✗

## Discussion

The present study aims to shed light on the complicated relationship between pitch perception and autism. Using various pitch perception tasks and a large sample of participants ranging from children to adults, we investigated whether: (i) autistic and NT individuals would differ in their pitch perception ability; (ii) pitch perception is related to cognitive abilities; and (iii) if so, whether cognitive abilities may modulate the relationship between pitch perception and autism. We also explored whether different types of matching procedure (group- vs. individual-level matching) common in autism research would result in different pattern of results.

Across all the pitch tasks and analyses used in the present study, we did not find any evidence of enhanced pitch perception among autistic relative to NT individuals. Instead, we found either similar or poorer performance by autistic individuals, as summarised in Table 3. There is no evidence of any subgroups within each group either, given similar distribution of high performers in each task. The lack of group differences in some of the pitch tasks when participants were matched on cognitive abilities at the group-level is consistent with previous findings (Chowdhury et al., 2017; Germain et al., 2019; Globerson et al., 2015; Jones et al., 2009; Schelinski & Roswandowitz, 2017), and highlights the potential role of cognitive abilities, rather than autism diagnosis, that drives group differences in previous studies when they were not matched (Eigsti & Fein, 2013; Yu et al., 2015). Here, the present study additionally found that when participants were matched individually, autistic individuals may in fact have poorer performance than NT individuals on some pitch tasks, particularly those that do not use musical stimuli whereas similar performance as neurotypical participants were observed in pitch tasks that used musical stimuli. This suggests a preserved ability for musical pitch perception among autistic individuals, in line with the findings of a possible dissociation between music and non-music pitch perception ability among autistic individuals (Jiang et al., 2015; Schelinski & von Kriegstein, 2019). However, as the pitch tasks were not matched in their difficulties, this remains speculative and would require further investigation using a more systematic approach. Nonetheless, this seems a promising avenue to pursue, especially given the utility of music-based therapy (Simpson & Keen, 2011; Williams et al., 2021).

The present study also found the involvement of cognitive abilities on pitch processing, particularly when group-level matching analysis was conducted. This finding complements previous research that have previously linked various cognitive abilities and pitch perception among autistic and NT individuals (Chowdhury et al., 2017; Jamey et al., 2019; Kargas et al., 2015; Mayer et al., 2016). As can be seen in Table 4, intelligence and/or verbal memory appear to be involved in most of the pitch tasks used in the present study. While we have shown an association between cognitive abilities and pitch perception, the direction of the relationship remains unclear. Using intelligence as an example, it is not clear whether increased pitch (or, more generally, auditory) perception allows one to perceive more fine-grained stimuli and therefore gather more information, resulting in higher intelligence overall or whether higher intelligence allows one to excel at the tasks used to assess pitch perception. Thus, the specific role of each cognitive ability on the different pitch tasks and the direction of the relationship warrants closer examination in future studies. Another pattern observed in Table 4 is that, across groups, more cognitive abilities are involved as the tasks and/or the stimuli become more complex when matched individually, which likely reflects the different task requirements and therefore the strategies used by participants. It should be noted, however, that this finding may not capture pitch perception in all autistic individuals, given that all our participants have typical cognitive abilities (e.g., those with atypical cognitive abilities may show differential performance relative to NT individuals as task complexity increases). This is especially the case in the real world as pitch perception is likely to be even more complex than the tasks used in the lab. Thus, to further explore the relationship between pitch perception, autism, and cognitive abilities, further studies should recruit four groups of participants: those with and without autism, and within each, with and without atypical cognitive abilities.

Having established that certain cognitive abilities are involved in pitch perception, it is reasonable to ask whether it may modulate the group differences in pitch perception. Researchers have long recognised the importance of taking an individual differences approach in autism research – that is, to investigate the association of other background measures on the dependent variable beyond group membership (Jarrold & Brock, 2004). Yet, this is rarely done in previous pitch perception studies that compare autistic and NT individuals, presumably due to the small sample size in those studies. Echoing Jarrold and Brock’s (2004) conclusion, we argue that to truly understand the relationship between pitch perception among autistic and NT individuals, we need to consider how individual differences in their cognitive abilities might affect their pitch perception. By comparing the reduced models vs. the full models for each pitch task (as summarised in Table 5), we observed that when there was a group difference in the reduced models, this group difference disappeared (Pitch Direction in the individual-level analysis and Speech Discrimination in the individual-level analysis) when cognitive predictors were added in the full models, which suggests that cognitive abilities, rather than autism diagnosis, may be driving the group difference between autistic and NT individuals in those pitch tasks. In some cases, no group differences were observed in the reduced models, but were nonetheless revealed in the full models as interactions with cognitive predictors (Pitch Detection in both group- and individual-level analyses, and Piano Discrimination in the individual-level analysis). These findings suggest the different involvement of cognitive abilities between groups, which is consistent with several previous findings (Jamey et al., 2019; Kargas et al., 2015; Mayer et al., 2016). Overall, these findings highlight the importance to consider cognitive abilities in pitch perception when examining group differences even when the groups are matched.

Our findings have implications for other autistic experiences. Given that there is generally either no group difference or an impaired performance on pitch perception (when cognitive abilities were not considered), this may have a cascading effect on other higher-level tasks such as decoding affective and linguistic prosody (Globerson et al., 2015; Wang et al., 2021). Moreover, it is unlikely that autistic individuals’ auditory hypersensitivity is purely physiological in nature (Stiegler & Davis, 2010), at least for pitch perception, as our sample of autistic individuals did not show enhanced pitch sensitivity across the different tasks. Instead, psychological differences such as how the sounds are interpreted may partly contribute to autistic individuals’ auditory hypersensitivity.

Throughout the manuscript, we assume that autism diagnosis is the underlying cause of our findings. However, autistic individuals often have other co-occurring conditions such as attention deficit hyperactivity disorder (ADHD) (Simonoff et al., 2008) that may also partly contribute to our findings given that the pitch tasks used in the present study do require participants to maintain their attention throughout the task (though note that participants were given opportunities for breaks within each task). In our sample, six autistic participants also reported having ADHD while none of our NT participants did, and so we reran the analyses without those participants in the group-level sample and without those participants and their matched controls in the individual-level sample. We found that, by and large, the findings were similar: though there were some changes in the significance of the predictors, most do not involve the Group variable except for MBEA in the individual-level analysis (after removing the ADHD participants, the Group effect was significant in the reduced and full models such that NT participants’ performance was significantly better in the MBEA task). Given that the stimuli for MBEA are much longer in duration than the other pitch tasks in the present study, this suggests a possible contribution of attention in addition to autism in pitch perception for longer stimuli, which is worth further investigating in future studies. Importantly, when the findings across all the pitch tasks are considered as a whole, these differences do not fundamentally change our general findings that (i) there is no enhancement in pitch perception among autistic individuals; (ii) cognitive abilities, in particular, Intelligence and Verbal Memory, are associated with pitch perception; and (iii) cognitive abilities modulate the relationship between autism and pitch perception for certain pitch tasks (see Supplementary Section S5 for a summary of the findings similar to Tables 3, 4 and 5 after excluding participants with ADHD and their matched controls).

While it is evident that matching participants is important for case-control studies, it is not clear whether there would be any difference in findings if participants were matched at the group- or individual-level. As a secondary objective, we examined this by analysing our results on two sets of samples: one in which participants were matched overall as a group and another in which participants were matched individually. Generally, our findings showed that when matched at the individual level, there is no increase in the likelihood to detect group differences (both approaches revealed group differences in 2 out of 5 pitch tasks; see Table 3) but a decreased likelihood of revealing the importance of other predictors (see Table 4). Assuming sufficient statistical power, we speculate that individual-level matching likely results in a ‘purer’ comparison between groups while limiting the variability in the other predictors. Indeed, one issue with matching in case-control design, as argued by some, is that matching may invariably introduce a bias by making the groups ‘too similar’ (Pearce, 2016). In a similar vein, comparisons of AIC values to examine model fit revealed that whereas the full model (i.e., with all the cognitive predictors) was preferred in most of the pitch tasks (4 out of 5) for group-level matching, the reduced model (i.e., without any cognitive predictors) is preferred in many tasks (3 out of 5) for individual-level matching. This suggests that when matched individually, the explanatory power of the other predictors is reduced, presumably due to the groups being more ‘similar’. Based on this, for future studies that employ a case-control design, we suggest that the matching procedure used should balance between addressing the research question and having sufficient resources. For example, if the study takes an individual differences approach, then individual-level matching may not be sensitive enough unless there is a large sample size, which may not be feasible to implement for some studies.

### Limitations

In addition to the future directions highlighted in the previous section, future studies should also address several limitations of the present study. Firstly, the autistic participants in the present study represent a subgroup of autistic individuals only, given that our autistic participants did not have any intellectual or verbal impairment, and so our findings may not be applied to those with intellectual or verbal impairment. Of the handful of studies that have investigated pitch perception ability of autistic individuals with intellectual or verbal impairments, the results are not conclusive. For example, in a previous study, a subgroup of three autistic participants were found to have the highest pitch memory and discrimination scores, and these three participants had low vocabulary scores (scoring about 1 standard deviation below the group mean) and one participant had a low full-scale IQ score (scoring a standard score of 42) (Heaton, Williams et al., 2008). Thus, it is possible that those with intellectual and/or verbal impairments may show enhanced, instead of impaired, pitch perception ability relative to other autistic individuals, but this would need to be confirmed with more data. Our criterion of excluding those with intellectual disability in our sample has also limited our opportunity to study musical savants with below normal IQ (Mottron et al., 1999; Young & Nettelbeck, 1995) who may provide an interesting insight on how pitch is processed among autistic individuals. Secondly, while we have observed age-related improvements in all the pitch tasks, the cross-sectional design of the study limits our interpretation of whether the developmental trajectory of improved pitch ability between autistic and NT individuals are similar. Longitudinal data would be required to establish this conclusively. Finally, future studies could also address the issue of not having participants complete all the tasks, resulting in an unequal sample size across the pitch tasks. Our initial plan was to perform a principal component analysis on all five pitch tasks to extract the components of pitch ability, but this would require each participant to complete all the pitch tasks. This study is a part of a larger project, and so some pitch tasks were introduced later than others, resulting in only some participants completing all the pitch tasks. Note, however, that, the smallest sample size in the present study (*n* = 25 in each group) is comparable to those of previous studies.

## Conclusions

In conclusion, across various pitch tasks and a wide age range, we did not find any advantage among autistic individuals on pitch perception relative to NT individuals—the former’s performance was either similar or poorer than that of the latter. We also found that certain cognitive abilities, particularly Intelligence and Verbal Memory, were associated with the pitch tasks. Importantly, the cognitive abilities modulated the relationship between autism and some of the pitch task performance: on some tasks, the group differences either disappeared or interacted with cognitive abilities when they were included in the statistical models. As a secondary objective, we also examined whether the findings would differ depending on matching procedures. While we were not more likely to observe group differences when participants were matched individually, we were less likely to observe the contribution of other predictors when matched at the individual level. This study has thus demonstrated the importance of considering cognitive abilities when investigating the complex relationship between pitch perception and autism and, more generally, the importance of taking an individual differences approach to understand the strengths and weaknesses of autism.

## Data Availability

The dataset supporting the conclusions of this article is available in the University of Reading Research Data Archive, 10.17864/1947.316.

## Footnotes

1 In this paper, we use the term ‘autistic individuals’, a term that is preferred by most autistic individuals and their family, to refer to individuals with ASD (Kenny et al., 2016).

2 Though rare, in cases where there might be multiple control matches for a case, we selected the control that best matched the case in their cognitive scores.

3 As our sample spans a wide age range, their autism diagnosis may differ based on the diagnostic criteria of that time. In our sample of autistic participants, 32 received a diagnosis of Asperger Syndrome, 26 received a diagnosis of Autism Spectrum Disorder, and only one received a diagnosis of Pervasive Developmental Disorder-Not Otherwise Specified (PDD-NOS). Excluding the PDD-NOS participant, the other two autistic subgroups, however, did not differ in their performance on any of the pitch tasks used (see Supplementary Section S1); thus, similar to what has been done in previous studies (Bonnel et al., 2003; Heaton, 2005), we grouped the autistic subgroups as a single autism group.

4 AQ scores from two NT participants were not available.
